# MoS_2_ Nanoparticle Effects on 80 °C Thermally Stable Water-Based Drilling Fluid

**DOI:** 10.3390/ma14237195

**Published:** 2021-11-25

**Authors:** Mesfin Belayneh, Bernt Aadnøy, Simen Moe Strømø

**Affiliations:** 1 Department of Energy and Petroleum Engineering, University of Stavanger, 4036 Stavanger, Norway; 2 Equinor ASA, Forusbeen 50, 4035 Stavanger, Norway; simen.stromo@hotmail.com

**Keywords:** thermal stability, lignosulfonate, molybdenum disulphide nanoparticle

## Abstract

Bentonite-based drilling fluids are used for drilling, where inhibitive fluids are not required. The rheological and the density properties of the drilling fluids are highly affected by high temperature and pressure. Due to high temperature, the clay particles stick together, and the fluid system becomes more flocculated. Poorly designed drilling fluid may cause undesired operational issues such as poor hole cleaning, drill strings sticking, high torque and drag. In this study, the 80 °C thermally stable Herschel Bulkley’s and Bingham plastic yield stresses drilling fluids were formulated based on lignosulfonate-treated bentonite drilling fluid. Further, the impact of a MoS_2_ nanoparticle solution on the properties of the thermally stable base fluid was characterized. Results at room temperature and pressure showed that the blending of 0.26 wt.% MoS_2_ increased the lubricity of thermally stable base fluid by 27% and enhanced the thermal and electrical conductivities by 7.2% and 8.8%, respectively.

## 1. Introduction

Global energy demand is increasing rapidly due to population growth and industrial activities. The petroleum industry is expanding exploration activities in the deep-water and arctic regions. Moreover, green energy exploration such as geothermal energy is receiving lots of interest, where one can extract thermal energy from the high-temperature formation. However, it is evident that inventions of improved technologies are required to handle high pressure and high temperature (HPHT) conditions.

Drilling fluids are an essential part of drilling operations, which have several functions such as to maintain well pressure, cool and lubricate the well, and bring cuttings up to the surface. The most important considerations for the selection of drilling fluids are their performance, cost, and environmental impact [1,2]. Oil- and water-based drilling fluids have been used extensively in drilling operations. In terms of performance, oil-based mud (OBM) has some fundamental advantages over water-based mud (WBM). This includes high drag reduction, shale swelling inhibition, and temperature stability [3]. However, OBM has environmental concern, disposal issue, and problem on health and safety [4,5]. For instance, the application of OBM is not allowed in environmentally sensitive area such as in North Sea [6]. Since OBMs do not contain polymers like in the WBM, barite sagging is also an issue due to insufficient gel structure formation [7]. 

Drilling fluids are formulated with density, viscosity, and filtrate loss control additives to achieve the desired drilling fluid’s physical and rheological properties. Bentonite is the common additive used to provide viscosity and gel strength of the water-based drilling fluid. In the presence of electrolyte, or other flocculating compounds, bentonite particles may flocculate due to the net force between the negative charge on the surface of the plates and the positive charge of the edge of the bentonite or ions in the systems [8]. Additionally, at an elevated temperature, bentonite particles adhere to each other and increase the gelation of the drilling fluid [9]. The high temperature changes the drilling fluid’s properties, such as an increase filtrate loss as a result of poor network structure and a higher viscosity due to water loss [10].

For efficient and safe drilling operations, it is imperative to design a drilling fluid that withstands the high temperature and pressure of the drilling environment. There are several thinning/deflocculating agents such polyphosphate, *quebracho*, tannate, Q-Broxin, lignosulfonate, and lignite [1]. Mixing dispersant (e.g., lignosulfonate) with the flocculated system, the dispersant will be adsorbed at the edges of clay particles. The balance forces acting on these clay particles changes from an attractive force to a repulsive force and the system will then be deflocculated. In addition, lignosulfonate stabilizes emulsion of oil-well drilling fluid by creating electrokinetic charge and a semirigid film at the oil and water interface [11].

Polymers are used in water-based drilling fluids to provide viscosity and control filtrate loss. Polymer type, concentration, surface chemistry (ionic and non-ionic), and molecular weight as well as its interaction with other drilling fluids ingredients determines the rheological properties, filtrate loss and thermal stability of the drilling fluids [12]. Nevertheless, higher temperature has the effect of breaking or associating polymer chains that results in a reduction of the viscosity [13]. Even though dispersant controls the deflocculating of the bentonite, water-based drilling fluids still have drawbacks in comparison with the oil-based drilling fluids [3]. This indicates the potential to improve the properties of the water-based drilling fluids.

In recent years, the application of nanotechnology (1–100 nm) has shown impressive performance in the oil and gas industry. The surface area to volume ratio of nanoparticle is higher than the microparticles [14]. Several nanoparticle-based laboratory experimental results reported that the nanoparticles improve the performance of the conventional water-based-and oil-based drilling fluids. The impact of the nanoparticle on the drilling fluids depends on the atomic structure, the morphology, the concentration, and the surface chemistry interaction with the base fluid’s chemical ingredients both physically and chemically. Among the documented research results, the blending of nanoparticles with water-based drilling fluid enhances the rheological properties of drilling fluid [15,16,17,18], reduces filtrate loss and the filter cake thickness [15,16,17,18,19,20], reduces the permeability of the shale by plugging the pore spaces [21,22,23], reduces the coefficient of friction [16,17,24,25,26], increases the wellbore strength [27], enhances the electrical conductivity and thermal conductivity of the conventional drilling fluids [28,29,30,31,32], and inhibits shale swelling [20,33,34].

From the reviewed literature studies, it is observed that a single nanoparticle does not enhance the rheological, thermal, electrical, filtrate loss, physical and other desired properties of drilling fluid. The impact of nanoparticles also varies in different base fluids. For instance, nanoparticles may be effective in improving the drilling fluid’s lubricity, but it may not have impact on the viscosity, the filtrate loss and shale swelling inhibition characteristics. The main reason is that different nanoparticles have different surface charge, thermal, electrical, atomic bonding and structural make up as well. As a result, the nanoparticle’s chemical/physical interaction with the base fluids are the determining factors on the behavior of the drilling fluids.

In this paper, the effect of MoS_2_ nanoparticles was evaluated on the 80 °C thermal stabile bentonite-based drilling fluid, which was formulated with pH, fluid loss, dispersant, viscosity and weight control materials. Negatively charged nanoparticles may have a dispersant effect in the bentonite drilling fluid. However, formulating bentonite drilling fluid with lignosulfonate (LS) dispersant will reduce the concentration of nanoparticles. By doing this, cost and nanoparticle-related environmental issues will be reduced considerably. Molybdenum disulphide has been used as lubricant in several industries such as automotive fields [35]. The main reason for the selection of the particle in this paper is due to its low frictional property and to investigate if the particle may have a multifunctional effect on improving the WBM properties.

## 2. Materials and Methods

### 2.1. Materials

The chemical ingredient for the formulation of nanoparticle-free base-fluids such as viscosity control agents (Duo-Vis polymer, bentonite), weight material (barite) and dispersant (lignosulfonate) were obtained from a local service company, MI-SWACO (Stavanger, Norway). Anhydrous pH control chemical, soda, was purchased from Sigma Aldrich (Oslo, Norway).

An inorganic Molybdenum disulphide (MoS_2_) nanoparticle was used for the formulation of the nanobased drilling fluid. The 30 wt.% molybdenum disulfide nanoparticles in water solution were purchased from US Research Nanomaterials (Houston, TX, USA). According to the manufacturer, the particle’s Mohs hardness is 1 to 1.5 -and the friction coefficient is 0.03~0.05. The particle exhibited excellent oxidation resistance (i.e., chemical degradation) and high temperature strength [36]. The density of the particle is 4.8 g/cm^3^.

### 2.2. Characterization and Analyses Methods

#### 2.2.1. Scanning Electron Microscopy (SEM)

Scanning electron microscope (Supra 35VP model, Zeiss, Oberkochen, Germany) was used to analyze the structure and the deposition of MoS_2_ nanoparticle in the mud cake.

#### 2.2.2. Viscosity Measurements

A rotational viscometer, OFITE Model 800 (OFITE, Houston, TX, USA) and heating cup was used to measure the viscosity of the drilling fluids. The viscometer responses of the drilling fluids were measured at three different temperatures (22 °C, 50 °C, 80 °C) under atmospheric pressure condition at the rotational speeds were speeds of 600, 300, 200, 100, 60, 30, 6, and 3 revs/min (RPM). The rheological parameters are determined using rheology model Herschel–Bulkley [37] and Bingham Plastic models (API-13D) [38].

#### 2.2.3. Anton Parr Rheometer

An Anton Paar rheometer (MCR 302) (Anton Paar GmbH, GRAZ, AUSTRIA) was used to measure the viscoelasticity of drilling fluids. An oscillatory amplitude sweep dynamic loading was applied on fluid specimens placed between parallel plates with a constant angular frequency of 10 rad/s and varying % strain in the range of 5 × 10^−4^% to 1000%.

#### 2.2.4. API Filter Press

The filtrate loss of the drilling fluids was measured with the static API Filter Press (OFITE, Houston, USA) by applying 100 psi for 7.5 min at room temperature.

#### 2.2.5. CSM Tribometer

A CSM tribometer (CSM Instruments, Needham, Massachusetts, USA) with ball-plate setup was used to measure the lubricity of the drilling fluids. A 6 mm-diameter 13Cr steel ball was rotated at the speed of 3 cm/s for 10 m of 3 cm/s by applying a 5 N load on the ball-plate interface. The measurements were conducted at room temperature. From the repeat tests, the average values are reported in the paper.

#### 2.2.6. ICP-EOS Filtrate Element Analysis

The ionic concentration of the drilling fluids filtrate was quantified with inductively coupled plasma (ICP). The samples were filtered through a 0.45 µm filter, prediluted 1:5 and 1:20 with 5% HNO3. Both dilutions were analyzed by ICP-OES to determine the number of preselected elements in water and dissolved solid.

#### 2.2.7. Thermal Conductivity Analysis

The thermal conductivity of the drilling fluid was also characterized with Tempos (Thermal Properties Analyzer Meter, Decagon, Pullman, WA, USA). During testing, electric power raises the temperature of the probe wire. Tempos is programmed to calculate the thermal conductivity using the temperature gradient characteristic of the wire by using a transition hot wire method. Several readings were taken, and the average values are reported.

#### 2.2.8. Electrical Conductivity Analysis

A RS PRO Conductivity Meter (RS 1410-1002 model, Bad Hersfeld, Germany) measured the electrical conductivity of DI (deionized) water and saltwater. It has a range of 0.0 µS to 2000 µS/cm with an accuracy of ±0.5%. The instrument is autocalibrated and measures conductivity. Immersing the probe in the drilling fluid specimen provides an automatic measurement.

#### 2.2.9. Modelling of Viscosity Characterization Method

Among the non-Newtonian rheology models, Herschel–Bulkley [37] approximates the yield stress of the drilling fluid quite well. The model is a yielded power law model, which is described by three parameters as:

(1) $$\tau =\tau_{y}+k{\dot{\gamma }}^{n}$$

where, the shear rate ($\dot{\gamma }$) and the shear stress (*τ*) are the measured values. The flow index (*n*) and the consistency index (*k*) are determined by curve fitting once the yield stress (*τ_y_*) is estimated. Reference [39] proposed that the yield stress can be estimated from the lower viscometer measured reading data as

(2) $$\tau_{y}\left[\frac{lbf}{100sqft}\right]=\;2.\theta_{3}-\;2\theta_{6}$$

Several investigators have used the API Bingham Yield stress for the analysis of thermal stability drilling fluid [40,41,42]. The Bingham plastic model is described by plastic viscosity and yield stress as: [38].

(3) $$\tau =YS+PV\dot{\gamma }$$

The Bingham plastic yield stress (*YS*) and plastic viscosity (*PV*) can be determined from the higher viscometer measured reading data as:

(4) $$YS\left[\frac{lbf}{100sqft}\right]=\;2.\theta_{300}-\;\theta_{600\;}$$

(5) $$PV\left[cP\right]=\;\theta_{600}-\;\theta_{300\;}$$

### 2.3. Drilling Fluid Formulation

In this paper, a modified version of reference [43] water-based drilling fluid was synthesized, in which the effect of MoS_2_ nanoparticles to be evaluated. The modification was by adding dispersant (i.e., lignosulfonate) to obtain a thermally stable ‘flat rheology’. Furthermore, Xanthan gum polymer was replaced by Duo-vis biopolymer, the bentonite concentration increased by 0.4% and reducing the barite concentration resulted in 1.31 sg mud weight. Table 1 shows the recipe of the base fluid. Drilling fluids were mixed with a high-speed Hamilton beach mixer and aged for 48 h at room temperature to ensure that bentonite swelled sufficiently. The chemicals mixing was according to the procedure described in reference [43]. To determine the optimum concentration of dispersant that provides a stable base fluid, for the given 10 g benitoite base fluid, lignosulfonate was varied in the range of 0.0 wt.% to 0.39 wt.% by weight of the drilling fluid. The result shows that the 0.175 wt.% (i.e., 0.9 g) lignosulfonate was sufficient to maintain the thermal stability of the Herschel Bulkely and Bingham yield stress up to 80 °C. Therefore, the 0.175 wt.% lignosulfonate-based drilling fluid was selected as a reference base fluid. Three nanoparticle-based drilling fluids formulated by mixing the base fluid MoS_2_ nanoparticle. The concentration of the MoS_2_ nanoparticle is given by weight percentile (wt.%) of the base fluid.

## 3. Results and Discussion

### 3.1. Effect of MoS_2_ and Temperature on the Viscosity of the Thermal Stable Drilling Fluid

**Figure 1:**
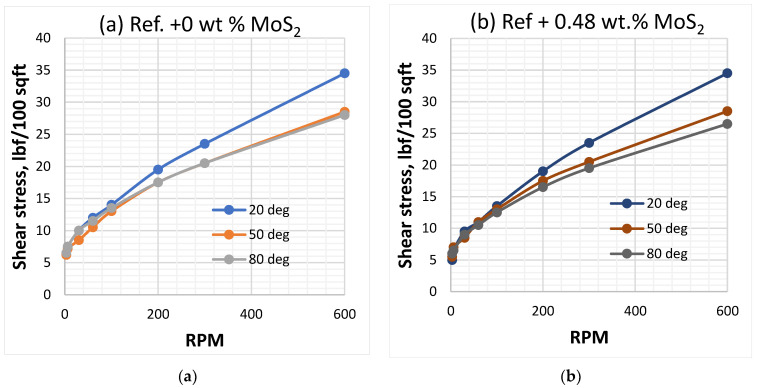

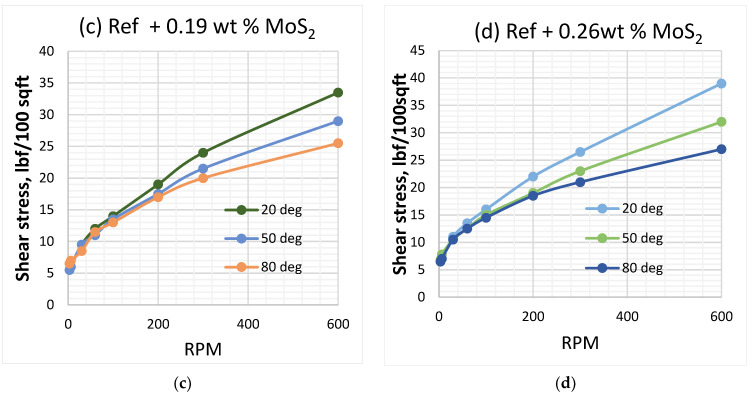
VG meter response of drilling fluids measured at (22 °C, 50 °C and 80 °C). (**a**) Reference-based fluid, (**b**) 0.09 wt.% MoS_2_ blended fluid, (**c**) 0.19 wt.% MoS_2_ blended fluid, (**d**) 0.26 wt.% MoS_2_ blended fluid.

Figure 1 shows the viscometer responses of the reference and MoS_2_ nanoparticles blended drilling fluids, which are measured at 20 °C, 50 °C and 80 °C. The viscosity of the drilling fluids decreased as the temperature increased and the effect is non-linearly with the concentration of the nanoparticle. One clear observation is that at the lower shear rate up to 100RPM, which is a typical drilling string rotational speed, the impact of the considered temperatures on the bentonite drilling fluids are insignificant. Additionally, the combined effect of nanoparticles and lignosulfonate maintain the bentonite platelets from being flocculated, which is due to the presence of the net repulsive electrostatic forces. However, at higher RPM, the viscometer responces deviate from the one measured at room temperature. 

### 3.2. Effect of MoS_2_ and Temperatures on the Rheological Properties of the Thermal Stable Drilling Fluid 

**Figure 2:**
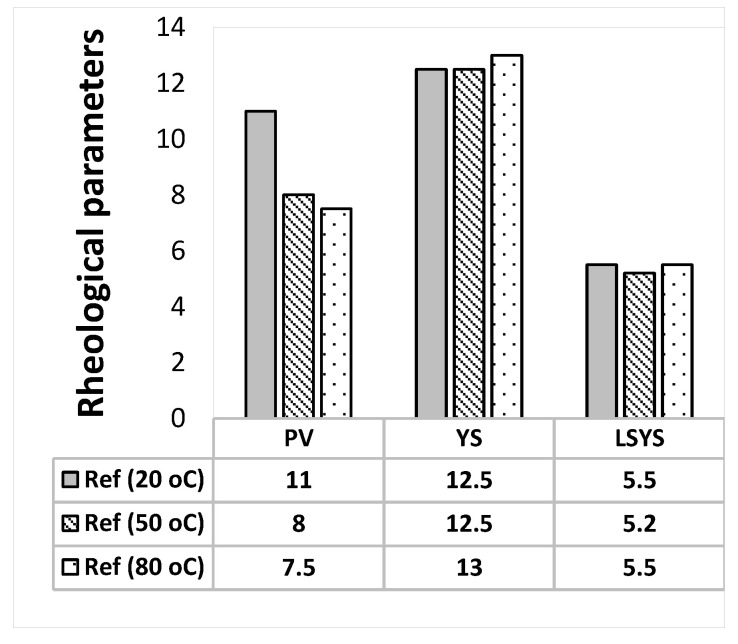
Reference MoS_2_-free drilling fluid.

**Figure 3:**
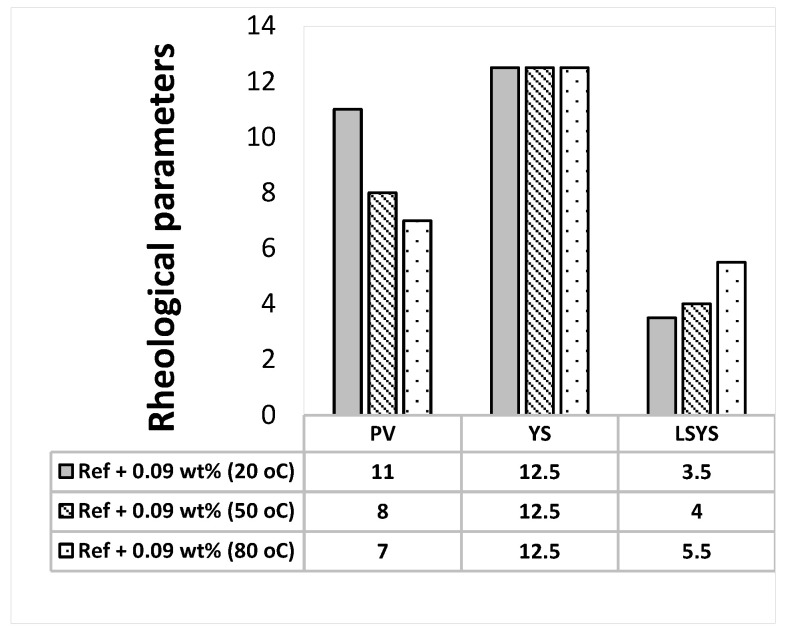
MoS_2_ blended drilling fluid—0.09 wt.%.

**Figure 4:**
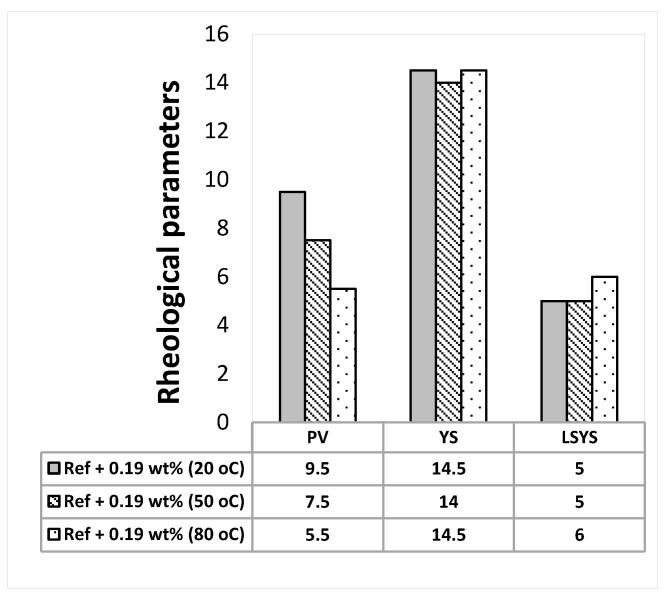
MoS_2_ mixed drilling fluid—0.19 wt.%.

**Figure 5:**
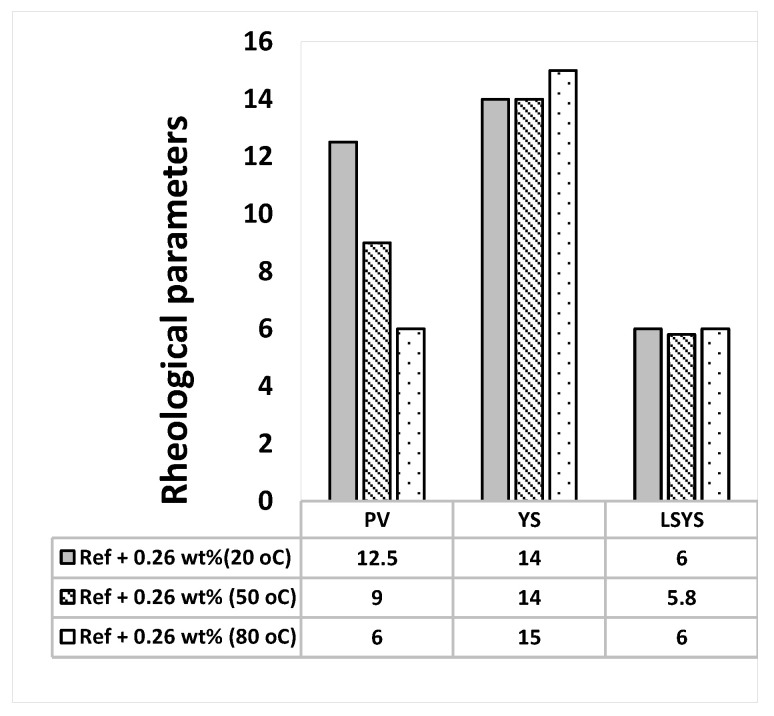
MoS_2_ mixed drilling fluid—0.26 wt.%.

The Herschel-Bulkley and the Bingham Plastic rheological parameters are calculated from the measured viscometer dataset and the results are presented in Figure 2, Figure 3, Figure 4 and Figure 5. As shown in the figures, the Herschel Bulkely yield stress and the Bigham yield stresses of the base- and MoS_2_ nanoparticle blended fluids are nearly thermally stable up to 80 °C. The magnitudes of the yield stresses are within the ranges of barite sag control recommended values [44]. On the other hand, the plastic viscosities decreased when the temperature increased. 

**Figure 6:**
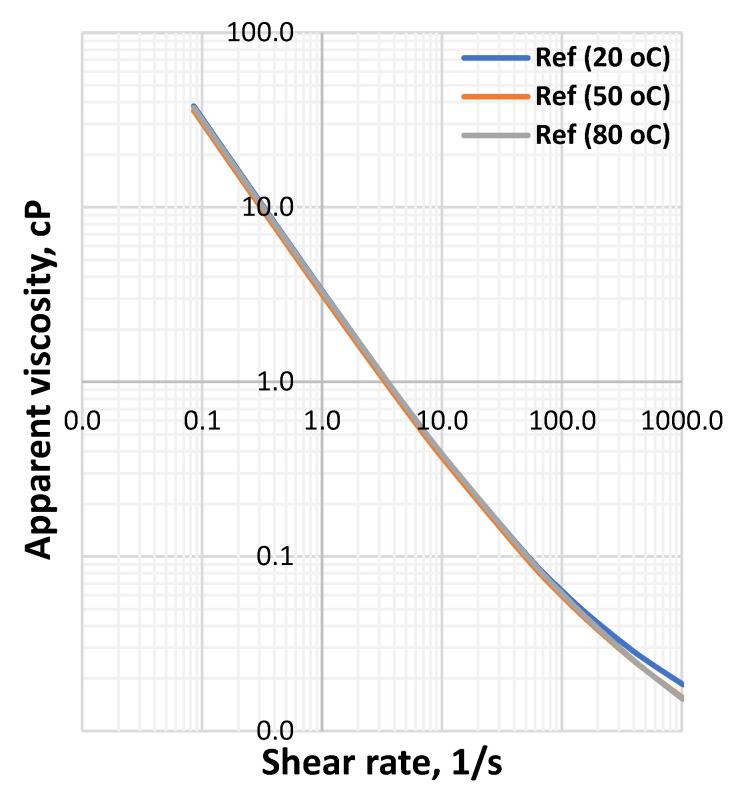
Reference MoS_2_-free drilling fluid.

**Figure 7:**
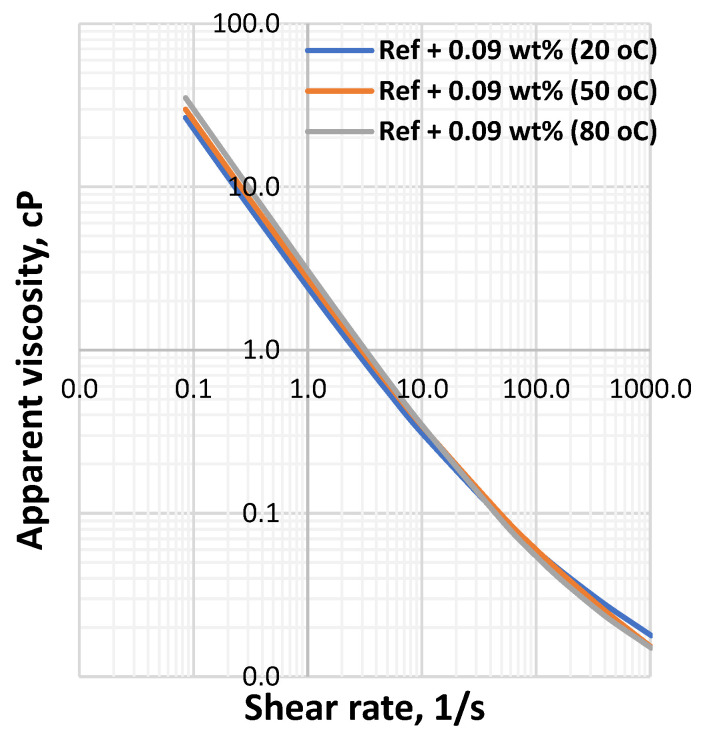
MoS_2_ blended drilling fluid—0.09 wt.%.

**Figure 8:**
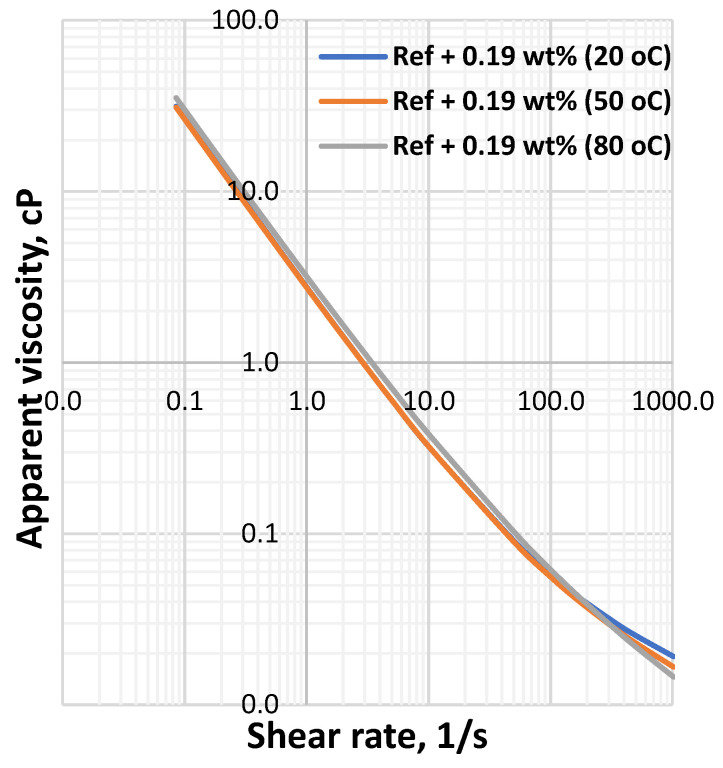
MoS_2_ mixed drilling fluid—0.19 wt.%.

**Figure 9:**
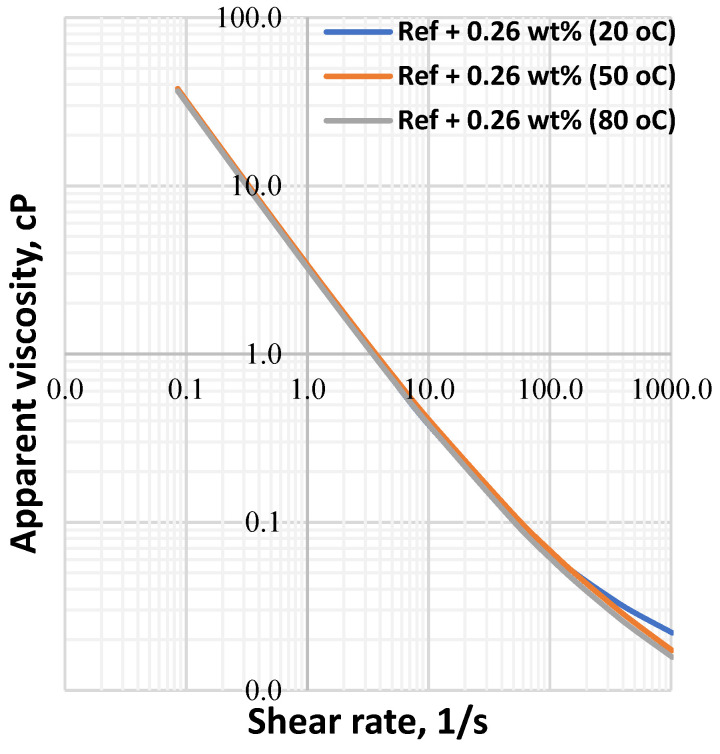
MoS_2_ mixed drilling fluid—0.26 wt.%.

The apparent viscosity a drilling fluid describes the behavior of the viscosity at the applied shear rate. To evaluate the combined effect of the Herschel Bulkely parameters (*τ_y_*, *n* and *k*), the apparent viscosity of the drilling fluids has been calculated and the results are displayed in Figure 6, Figure 7, Figure 8 and Figure 9 From the figures we can observe that the considered temperatures and nanoparticle concentrations show insignificant impact on apparent viscosity of the nano-free base fluid. This also shows the thermal stability behavior of the drilling fluids.

### 3.3. Effect of MoS_2_ on the Lubricity of the Thermal Stable Drilling Fluid 

The lubricity of the oil-based muds (OBM) generally higher than the water-based muds (WBM). It is therefore essential to enhance the lubricity of water-based drilling fluids to lubricate drill bit and hence reduce drill bit damage. Also, effective lubricant reduces the torque and drag issues, which allows to drill a longer drilling offset. 

**Figure 10:**
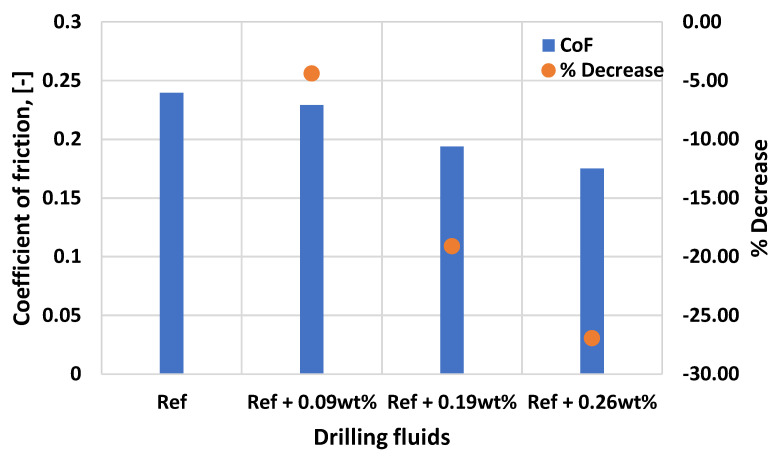
Effect MoS_2_ on the coefficient of friction of the reference base fluid.

Figure 10 shows the effect MoS_2_ on the coefficient of friction of thermal stable fluid system. As shown in the figure, the base fluid exhibits a lower coefficient of fiction, which could be in the presence of the lignosulfonate and Duo-vis polymers. However, the 0.29 wt.% MoS_2_ reduced the coefficient of friction of the base fluid by 27%. The reason for the enhancement is due to the forces that binds the MoS_2_ layers together are quite weak as compared with the covalent force according to reference [45]. Hence, MoS_2_ showed a lubricating effect because of the weak interlayer forces [46]. The particles contact with the ball-plate surface reduce the shear stress and hence resulting a less slippage resistance.

### 3.4. Effect of MoS_2_ on the Thermal Conductivity of the Thermal Stable Drilling Fluid 

Heat is generated on the bit due to bit and rock mechanical interaction. One of the main functions of the drilling fluid is to cool and lubricate the drill bit and the formation. Therefore, drilling fluids must be formulated to have adequate heat transfer properties in order to prevent drill bit overheating. Due to the large surface area, uniformly dispersed nanoparticles allow more heat transfer. Additionally, the mobility of the nanoparticles may bring about micro-convention of fluids and hence increase heat transfer [47]. Several investigators have studied the thermal behaviors of nanoparticles impact on drilling fluids such as (CNT [28], CuO, ZnO [29], CNT [31], SiO_2_, CNT, ZnO [30]) and their experimental investigations revealed that the nanoparticles enhanced the thermal conductivity of bentonite base drilling fluid. 

**Figure 11:**
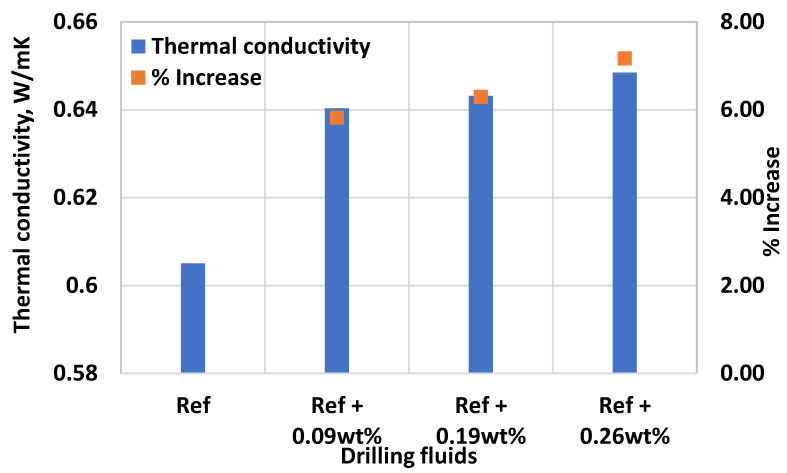
Effect of MoS_2_ nanoparticle solution on the heat conductivity of the base fluid.

Figure 11 shows the effect of MoS_2_ on thermal conductivity of thermal stable fluid system. For each fluid systems, several measurements have been performed, and the average values are reported. As shown in the figure, the MoS_2_ nanoparticles enhanced the thermal conductivity of the base fluid. As the nanoparticle’s weight percent increased from 0.09% wt.% to 0.26 wt.%, the thermal conductivity of the base fluid improved by 5.8 to 7.2% respectively. The thermal conductivity enhancement is linearly and gently as the concentration increases. According to reference [48], the reason for the improvement is due to the presence of more nanoparticles in the suspension causing the heat transfer enhancement between particles, and the micro convection enhancement between particle and base fluid.

### 3.5. Effect of MoS_2_ on the Electrical Conductivity of the Thermal Stable Drilling Fluid

**Figure 12:**
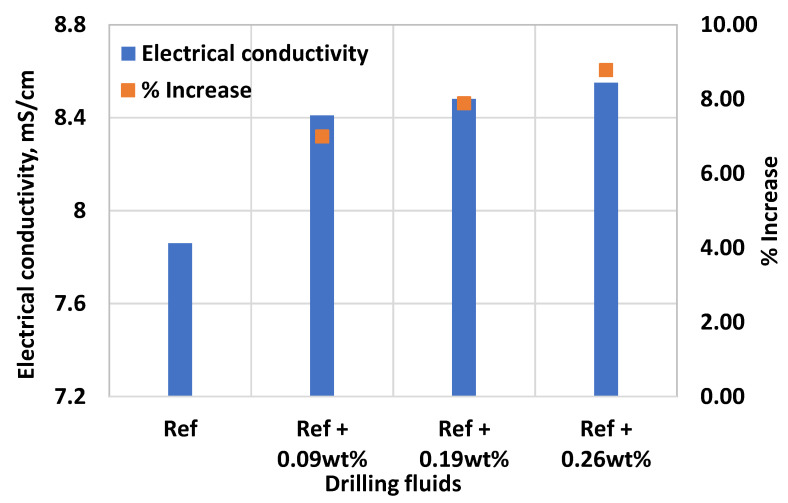
Effect of MoS_2_ nanoparticle on the electrical conductivity of the base fluid.

The electrical conductivity of the drilling fluid is also important property for downhole data transfer and the values should be within the desirable range for electrical logging operations. In this paper, the impact MoS_2_ on electrical conductivity of thermal stable fluid system have been measured and displayed in Figure 12. Results showed that 0.09 wt.%, 0.19 wt.% and 0.26 wt.% MoS_2_ nanoparticle concentrations increased the electrical conductivity of the base fluid by 7%, 7.9% and 8.8%, respectively. The mechanism for the enhancement of the electrical conductivity of the base fluid is due to the formation of electric double layer formed at surface charge of MoS_2_ nanoparticles [49]. The volume fraction of nanoparticle increases allow the availability of conducting path-ways that consequently increase the electrical conductivity of the drilling fluid [50]. Nanoparticles have high surface area and provides higher dispersion stability with principal Brownian motion of particles [51]. The electrical conductivity provides information about degree of dispersion and stability of the nanofluid [52]. Due to the high zeta potential of the MoS_2_ [36,53], the repulsive potential in the system is higher. The suspension then becomes stable and unlikely to agglomerate [50]. The increase electrical conductivity is therefore attributed to a good dispersion and suspension stability of the drilling fluid. In addition, the electrophoretic mobility of nanoparticles also reinforces effective electrical conductivity of the fluid system [50,51]. For interested readers, the theoretical electrical conductivity of nanoparticles due to the particle suspension, Brownian motion and electrophoretic mobility can be found from the references [50,51,54].

### 3.6. Effect of MoS_2_ on the Viscoelasticity of the Thermal Stable Drilling Fluid

**Figure 13:**
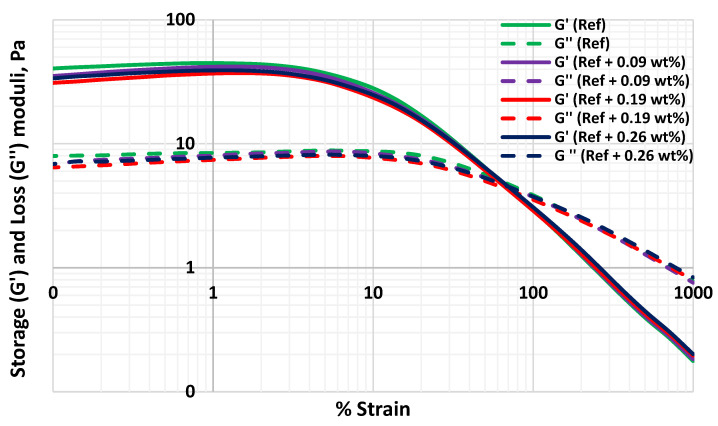
Dynamic amplitude tests of the drilling fluids.

Drilling fluids have the property of viscoelasticity [55,56]. Up on dynamic loading, energy store in the elastic part of a viscoelastic material and viscous part describes the loss of energy as heat during deformation. Figure 13 shows the amplitude sweep of the drilling fluids. As shown, despite the relative deviation of the storage and loss modulus are above and below the reference fluid, the storage to loss moduli ratio (damping factor) are nearly equal. 

For further characterization, the yield stress of the drilling fluid is determined from the dynamic amplitude sweep test. The deviation of the shear stress from the linear-elastic region represents the yield strength of the drilling fluid [57,58]. As shown in Figure 13, the flow point is the cross-point of the storage and loss moduli, where the drilling fluid behave as equal portion of elastic and viscous. Table 2 shows the estimated yield stress and flow point stress values of the drilling fluids. As shown, the nanoparticles impact on the viscoelasticity of the base drilling fluid is insignificant as has been observed on the yield stress parameter. One of the main reasons could be due to the presence of sufficient lignosulfonate and MoS_2_ nanoparticles kept the bentonite from being flocculated and maintained the system being dispersed. 

### 3.7. Effect of MoS_2_ on the Filtrate Loss of the Thermal Stable Drilling Fluid 

Drilling fluids that produce good filter cake on the wellbore prevent fluid loss. The quality of filter cake with respect to fluid loss and mechanical strength depends on the additives in the drilling fluids such as fluid loss control polymers as well as the packing of solids deposited in the mud cake. Several investigators have reported the impact of nanoparticles that reduce the filtrate loss such as (Fe_2_O_3_, [15], SnO_2_ [17] MWCNT, Al_2_O_3_, CuO, TiO_2_ [16,19], MWCNT, Graphene, [18], Fe_2_O_3_, [20]) and silica reduce the permeability of the shale by plugging the pore spaces [21,22,23].

**Figure 14:**
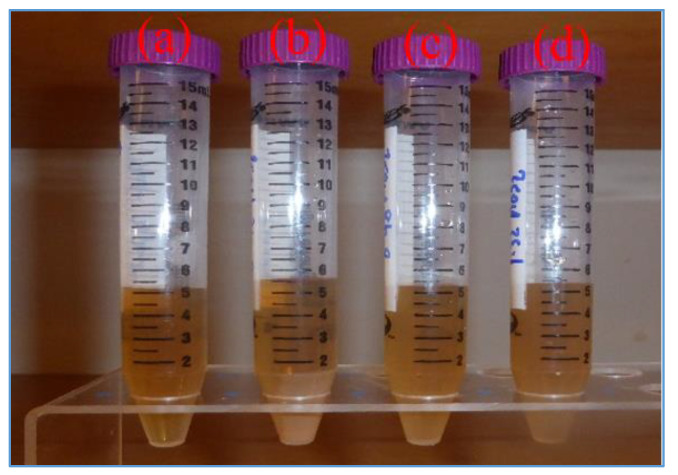
Drilling fluids (Filtrate loss): (**a**) Ref (5.2 mL), (**b**) Ref + 0.09 wt.% (5.5 mL) (**c**) Ref +0.19 wt.% (5.4 mL) (**d**) Ref + 0.26 wt.% (5.4 mL).

**Figure 15:**
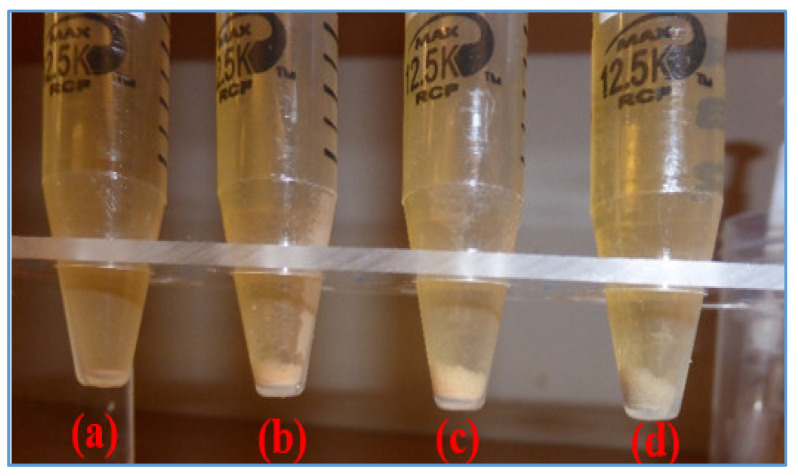
Solids precipitated in filtrates: (**a**) Ref (**b**) Ref + 0.09 wt.% (**c**) Ref + 0.19 wt.% (**d**) Ref + 0.26 wt.%.

Figure 14 shows the filtrate losses of the base -and MoS_2_ blended drilling fluids. As shown, the nanoparticle free base fluid exhibited 0.2-0.3 ml filtrate loss lower than the nanoparticle blended drilling fluids. At the bottom of the cylinders as displayed in Figure 15, solid particles are settled out of the filtrate. The solid concentration in the base fluid filtrate is less than the one in the nanoparticle blended filtrates. It can also be observed that the concentration of the precipitated solid increases with the increasing of the concentration of nanoparticles. The zeta potential of the MoS_2_ nanosheet possesses a higher number of negative charges [36,53]. The presence of MoS_2_ along with the lignosulfonate might have increased the electrostatic repulse forces, which resulted in more dispersion of bentonite palates or poor internal structure of the mud cake as well. It is interesting to observe that the nanoparticle concentration increment correlated with the volume of solids in the filtrate. During the synthesis of the drilling fluid, the blending of nanoparticles with the base fluid created more foams. About 6 drops of anti-foam were added to reduce the air bubbles. This could also be one of the reasons for the more filtrates in the nanoparticle based drilling fluids. 

**Figure 16:**
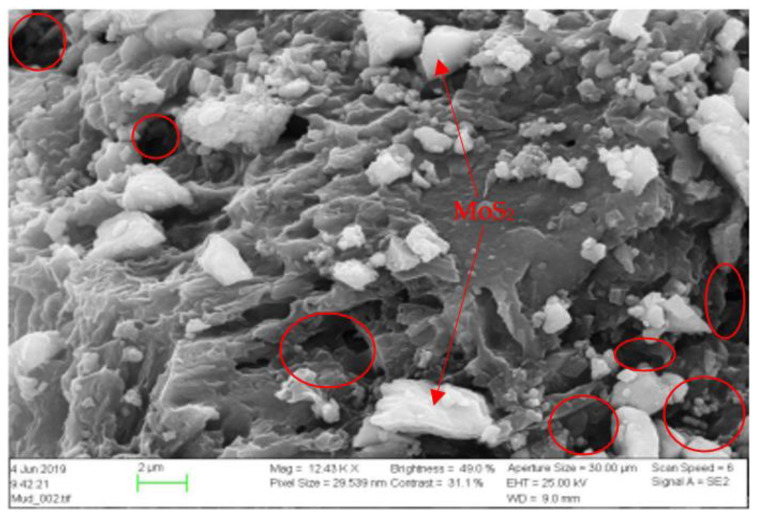
SEM picture of mud cake and MoS_2_ deposit (white particles).

Figure 16 shows the SEM picture of the 0.2 wt.% MoS_2_ based mud cake, which displays the internal and surface structure of the mud cake along with the nanoparticle’s placement. The particles are deposited on the surface and being plugs as part of the pore spaces. However, the mud cake also showed several nanoparticle unfilled pore spaces, through which filtrate loss can flow. In addition to the air bubbles presence in the drilling fluid, the porosity of the cake could the other possible reason for the more filtrate loss in the MoS_2_ blended fluid.

### 3.8. Effect of MoS_2_ on Filtrate Loss and Ionic Concentrations 

**Figure 17:**
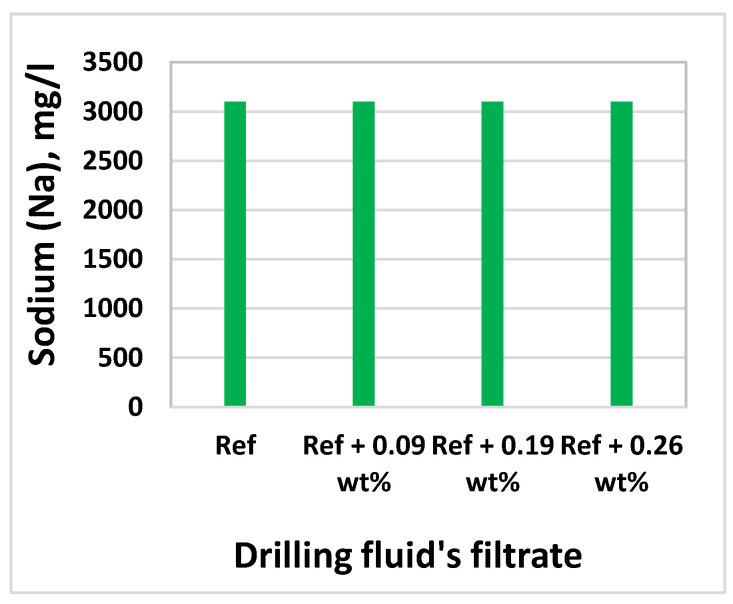
Na in the filtrates of drilling fluids.

**Figure 18:**
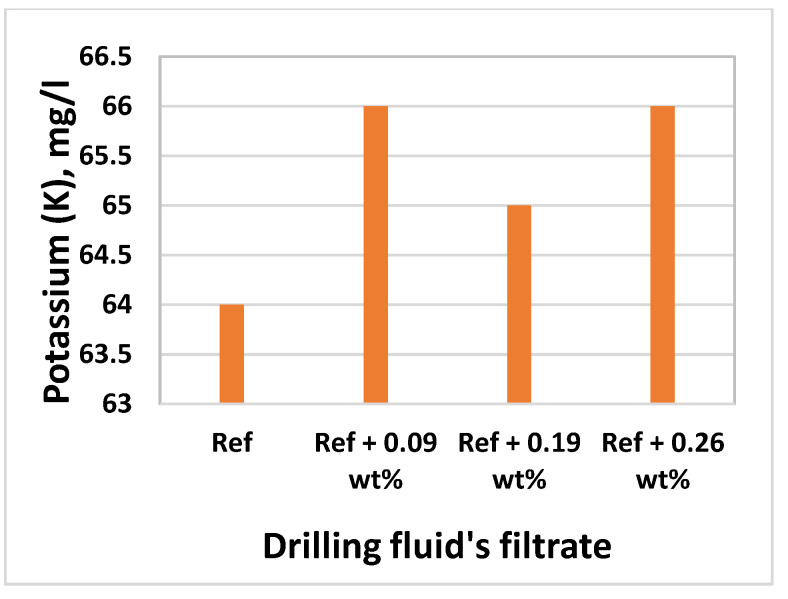
K in the filtrates of drilling fluids.

**Figure 19:**
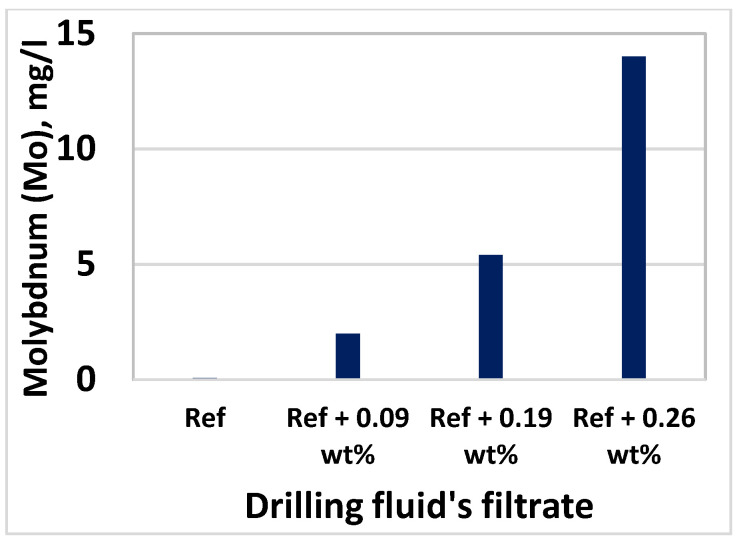
Mo in the filtrates of drilling fluids.

**Figure 20:**
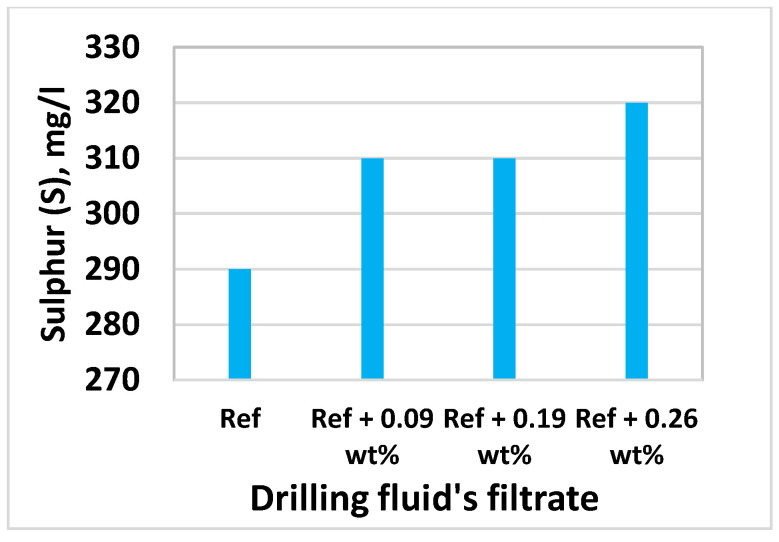
S in the filtrates of drilling fluids.

Inductively Coupled Plasma-Optical Emission Spectroscopy (ICP-OES) technique was used to determine the elements and concentrations that are existing in the filtrate. The analysis was conducted at InterTec Laboratory. Based on the drilling fluids chemical ingredients, we have selected Sodium (Na), Potassium (K), Molybdenum (Mo), and Sulphur(S) to analyze filtrate out - and deposition of ions on the mud cake. The element analysis results are displayed in Figure 17, Figure 18, Figure 19 and Figure 20 As shown, the amount of Na in the four drilling fluids is the same. The concentration of K detected in nanofluid filtrate is about 3.1% higher than the base fluid. This was due to the dissolved solids, in which potassium is part of the element in the barite. The concentration of molybdenum is the base fluid is 0.079 mg/l, which is obtained from Barite. As the MoS_2_ NPs concentration increases, the amount of Molybdenum and Sulphur also increase. As shown in Figure 16, the structure of the cake consists of more pore spaces, in which the nanoparticles flow through. The Sulphur elements comes from the lignosulfonate and the nanoparticles. Hence, Sulphur also detected in the base fluid filtrate about 6.9% less than the nanofluid filtrate. From the element analysis, we can observe that due to the higher repulsive force and the nature of the mud cake, the free smaller particles are able to flow through the filter paper.

## 4. Discussion

Drilling is a cost factor the oil and gas industry. Drilling activities becomes more complex in HPHT -and in depleted formation. Moreover, in an extended reach horizontal wells and in deep water depth, where the drilling operational window is narrow. In order to handle the narrow window and depleted formation operational challenges, the industry developed new drilling methods called managed pressure -and under pressure drilling methods, respectively. However, for conventional and the new drilling methods, the quality of the drilling fluid property is still essential. 

An efficient, cost effective and environmentally friendly drilling fluids are key for the successful drilling operation. Poorly designed drilling fluids results undesired operational challenges such as differential sticking, high drag, poor hole cleaning, and difficulty in well pressure control and resulting kick, well collapse and well fracturing [1].

Survey conducted on the European wells in 1989–2007 showed the increase drilling efficiency per day, which is due to an improved PDC bit technology. However, the study indicated a flat non-productive time that accounts about 25%] [59]. In deep-water and extended reach well, the NPT observed up to 40% [59]. Wells drilled Gulf of Mexico during 1993–2002 also showed that the non-productive time accounts about 24% [60]. The factors that contribute to the NPT are lost circulation, stuck pipe, kicks, wellbore instability, equipment failure, cement squeeze operations, directional control, weather delay and equipment handling. Among these, loss circulation, kick and stuck pipe account about one third of the overall NPT incidents [61]. These problems are in one or another way has connection with drilling fluids. It is therefore important to design good drilling fluids to minimized drilling fluid related undesired operational events. 

High temperature and high pressure influence the rheological and physical properties of the drilling fluids. As a results, the well pressure changes. In order to maintain the well pressure within the desired allowable drilling operational window, it is vital to design of the thermal and pressure resistance fluid. 

In this paper, at first, bentonite based thermal stable drilling fluid was formulated by varying the lignosulfonate concentration in bentonite drilling fluid. Lignosulfonate is an important additive when design bentonite base drilling fluid for geothermal and high temperature oil and gas wells to control undesired clay flocculation. The right concentration of the flocculant is determined through experimental works. Despite the thermal stability, water drilling fluids have a lower lubricity as compared with the Oil based drilling fluid. In order to improve the thermal stabile drilling fluid with other properties, nanoparticles have been treated. In literature as reviewed, nanoparticles have shown an improved performances on the drilling fluids. In this paper, MoS_2_ impact on the thermal stable drilling fluids enhances the lubricity, electrical and heat conductivity properties without impacting the thermal stability rheological behavior of the base fluids. In terms of filtrate losses, several investigators reported that different nanoparticle reduced the filtrate loss [15,16,17,18,19,20]. On the other hand, MoS_2_ used in this paper did not show any reduction impact. The observations indicate that the performance of nanoparticles in filtrate loss reduction depends on the chemistry of the nanoparticle’s interaction with the base fluid. In this paper, the lignosulfonate treated base fluid and the MoS_2_ are highly negatively charges and the system might have been deflocculated/dispersed well. Therefore, the performance of MoS_2_ with respect to filtrate loss is valid only in the base fluid formulated in this paper. However, MoS_2_ could reduce the filtrate loss in other base fluid. In the future, the effect of MoS_2_ will be evaluated in different commercial and in-house formulated base fluids. The effect of hot rolling will also be investigated.

The engineering implication of MoS_2_ on improving the lubricity and heat conductivity can be reflected in terms of allowing to drill longer offset by reducing the drag as well as cooling the bit in order to reduces early bit damage, respectively. 

## 5. Conclusions

Results from the experimental study showed that:

- The addition of 0.17 wt.% lignosulfonate maintains thermally stable H-B and Bingham plastic yield stresses.
- The 0.26 wt.% molybdenum disulphide additive enhanced the lubricity of the thermally stable base fluid by 27%. The nanoparticles maintain the thermal stability of the fluid and did not show a significant impact on viscoelasticity.
- Addition of 0.26 wt.% MoS_2_ nanoparticle solution increased the thermal and electrical conductivity of the base fluid by about 7.2% and by 8.8%, respectively.

To sum up, the impact of the MoS_2_ reported in this paper is valid only on the considered base fluid, the measurement temperature up to the 80 °C and atmospheric pressure conditions. By changing any one of these conditions, one may achieve different results.

## Figures and Tables

**Table 1:** Base fluid [Ref.] recipe without and with nanoparticle solution.

Chemical	Base Fluid Ref.	Nanofluid Ref. + MoS_2_
Water, [g]	350	350
Bentonite, [g]	10	10
DUO-VIS biopolymer, [g]	0.6	0.6
Anhydrous Soda Ash, [g]	3.2	3.2
Barite, [g]	150	150
Lignosulfonates, [g]	0.9	0,9
Nanoparticle (MoS_2_) [wt.%]	-	0.09, 0.19, 0.26
Anti-foam	6 drops	6 drops

**Table 2:** Yield and flow point of the drilling fluids.

Fluids	Yield Stress, Pa	Flow Point, Pa
Ref.	0.9	4.4
Ref. + 0.09 wt.% MoS_2_	1.0	4.5
Ref. + 0.19 wt.% MoS_2_	1.0	4.5
Ref. + 0.26 wt.% MoS_2_	1.0	4.5

## Data Availability

The data presented in this study are available on request from the corresponding author.
